# The Aortic Prosthesis and Aortic Valve Bioprosthesis Trombosis as a Late Complication in Patients after the Bentall Procedure Followed by a Valve-in-Valve Transcatheter Aortic Valve Implantation

**DOI:** 10.3390/diagnostics14182070

**Published:** 2024-09-19

**Authors:** Paweł Muszyński, Oliwia Grunwald, Maciej Południewski, Paweł Kralisz, Szymon Kocańda, Tomasz Hirnle, Sławomir Dobrzycki, Marcin Kożuch

**Affiliations:** 1 Department of Invasive Cardiology, Medical University of Bialystok, M. Skłodowskiej-Curie 24A, 15-276 Bialystok, Polandmarcin.kozuch@umb.edu.pl (M.K.); 2 Department of General and Experimental Pathology, Medical University of Bialystok, Mickiewicza 2C, 15-230 Bialystok, Poland; 3 Department of Cardiology, Lipidology and Internal Diseases, Medical University of Bialystok, Żurawia 14, 15-569 Bialystok, Poland; 4 Department of Cardiac Surgery, Medical University of Bialystok, M. Skłodowskiej-Curie 24A, 15-276 Bialystok, Poland

**Keywords:** cardiovascular imaging, ViV TAVI, valve thrombosis, aortic dissection, vascular complication

## Abstract

**Background:** Valve-in-Valve (ViV) transcatheter aortic valve implantation (TAVI) has emerged as a viable therapeutic option for structural valve degeneration following surgical aortic valve replacement (SAVR) or prior TAVI. However, the understanding of long-term complications and their management remains limited. **Case presentation:** We present the case of a 69-year-old male with a history of ViV-TAVI, who presented with symptoms of non-ST elevation myocardial infarction (NSTEMI) and transient ischemic attack (TIA). Computed tomography (CT) revealed thrombosis of the ascending aortic graft and aortic valve prosthesis. Transthoracic echocardiography (TTE) further confirmed new valve dysfunction, indicated by an increase in the aortic valve mean gradient. Treatment with low-molecular-weight heparin (LMWH) resulted in partial thrombus resolution. The multidisciplinary Heart Team opted against coronary angiography and recommended the long-term administration of vitamin K antagonists (VKAs). Follow-up CT showed the complete resolution of the thrombus. **Conclusions:** Thrombosis of the aortic graft and aortic valve following ViV-TAVI may be attributed to alterations in blood flow or mechanical manipulations during the TAVI procedure, yet it can be effectively managed with VKA therapy. CT is a valuable tool in coronary assessment in patients with NSTEMI and aortic valve and/or aortic graft thrombosis.

**Figure 1:**
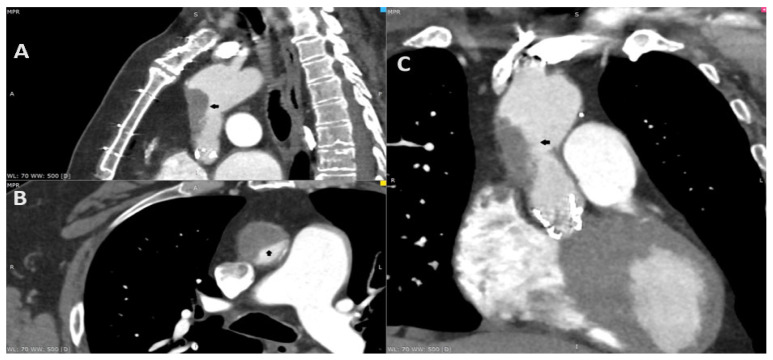
A 69-year-old male complaining of recurring chest pain, episodes of syncope and visual distortion was admitted to the hospital. His past medical history included the replacement of the ascending aorta with surgical aortic valve replacement (SAVR) in 2011 due to aortic aneurysm and aortic stenosis (Bentall procedure). He underwent transcatheter aortic valve implantation (TAVI) in 2021 due to the degeneration of aortic valve bioprosthesis, using a 26 mm balloon-expandable Sapien 3 Ultra (Edwards Lifesciences, Irvine, CA, USA). The treatment course after TAVI involved longitudinal single antiplatelet therapy (SAPT)—aspirin. In addition, he reported reduced exercise tolerance since the TAVI procedure. The coronarography in 2011 and angio-CT in 2021 excluded the significant coronary artery disease before operations. The angio-CT, including the aorta and head, at the current hospitalization found the thrombosis of the ascending aorta graft causing 80% stenosis (**A**–**C**; black arrow). Furthermore, due to the resolution of the neurological dysfunction, the patient was diagnosed with a transient ischemic attack (TIA). The transthoracic echocardiography revealed an increase in gradient through aortic valve bioprosthesis—a mean gradient of 24 mm Hg. The low-molecular-weight heparin (LMWH) in therapeutic dose was initiated. Despite the increase in cardiac enzymes, the chest pain and the diagnosis of non-ST elevation myocardial infarction (NSTEMI), the coronarography was not performed due to the risk involving the dislocation of the thrombi. After three days, the control CT displayed a significant reduction in the thrombus. The patient consulted with the Heart Team and was transferred to a higher reference clinical hospital with the availability of the cardiac surgery department.

**Figure 2:**
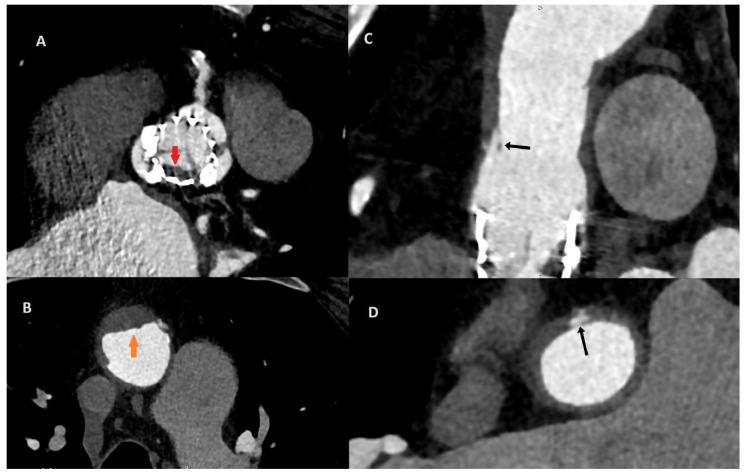
After the consultation with the cardiac radiologist, the third ECG-gated cardiac CT was performed on the eighth day after the initial CT to access both the aorta and coronary arteries. To increase the quality of the image, an additional beta-blocker and nitroglycerin were administered before examination. The CT revealed aortic valve prosthesis thrombosis (**A**; red arrow) and a reduction in aortic thrombosis (**B**; orange arrow). The previous thrombus site was localized to originate within the ascending aorta graft, which was covered by soft tissue that resembled an ulcer. The probable dissection of the neointima along the prosthesis was the suspected cause of the initial aortic thrombosis (**C**,**D**; black arrow).

**Figure 3:**
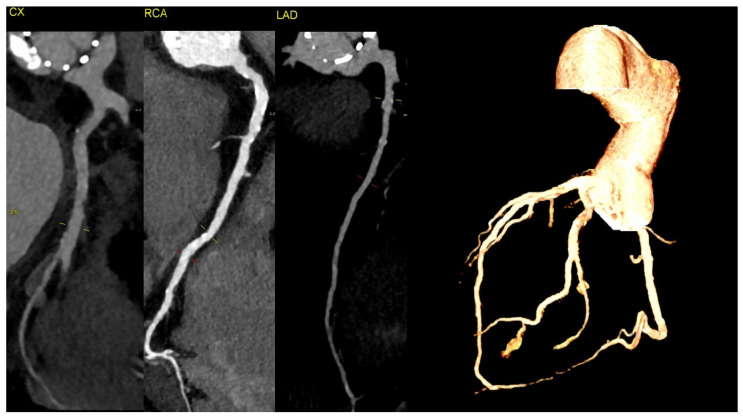
The CT discluded significant coronary artery stenosis (Cx—circumflex artery, RCA—right coronary artery, LAD—left anterior descending artery)**.** The myocardial infarction was treated conservatively with anticoagulation due to the presence of aortic graft and valve thrombosis, and it led to the resolution of the chest pain. The clinically insignificant lesions in the coronary arteries on CT were suggestive of the fact that NSTEMI could be caused by the occlusion of the ostium of the arteries by the aortic valve thrombus.

**Figure 4:**
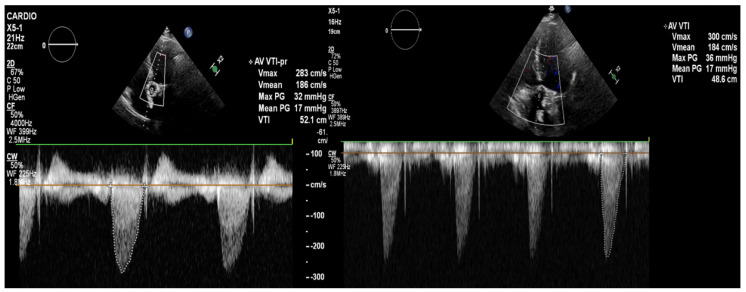
The transthoracic echocardiography showed a reduction in the gradients through the aortic valve prosthesis with parameters similar to that after the TAVI (on left: post-TAVI; on right: post-LMWH treatment). According to the 2021 ESC/EACTS Guidelines, in patients after TAVI, lifelong SAPT is recommended in the lack of indications for oral anticoagulants (OACs) [1]. However, despite similar recommendations for aspirin, the 2020 ACC/AHA Guidelines suggest that in patients with a low bleeding risk, it is worth acknowledging the antithrombotic prophylaxis with DAPT or VKA [2]. The direct-acting oral anticoagulants (DOACs) were not found to be superior to the administering antiplatelet or VKA, and in patients without indications for OAC, were linked to a higher incidence of all-cause mortality [3,4,5]. The 2021 ESC/EACTS and 2020 ACC/AHA Guidelines for managing valvular heart disease indicate that anticoagulation using VKAs or UFH is a first-line therapy for bioprosthetic valve thrombosis. Such an approach is highly effective in the normalization of valve function in 85% of the patients [6].

**Figure 5:**
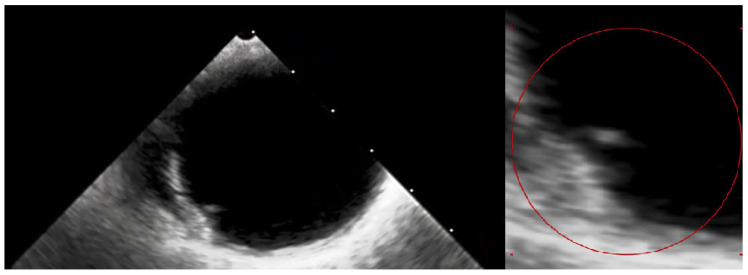
The transesophageal echocardiography failed to assess the aortic valve due to the artefacts. However, it confirmed the suspicion of the visible ruptured neointima within the aorta on the CT (Video S1—red circle). The patient again consulted with the Heart Team, and the decision about a conservative approach and administration of oral anticoagulation with vitamin K antagonists (VKAs) was sustained. At the 3-month follow-up, the patient was free from angina and syncope, and the control chest CT showed no sign of thrombus presence in the aortic region. The anatomical changes, such as the dimensions and shape of the aortic root, which occurred during TAVI, may have caused the dynamic switch in blood flow through the bioprosthetic valve, thus causing aortic remodelling. This contributes to the fact that the blood stagnates in the prosthetic sinuses, which complements Virchow’s triad and is attributed to thromboembolic events [7]. Furthermore, the ongoing structural bioprosthesis degeneration leading to fibrosis and calcification is often started by leaflet thickening and valve thrombosis [8]. The atherosclerotic plaque in the ascending aorta, or dissection of the neointima along the prosthesis, could also contribute to the thrombotic event. Aortic dissection was reported to be a rare TAVI procedure complication, occurring almost entirely as an acute condition. Regardless, there have been no previous reports of dissection of the neointima in the aortic prosthesis after ViV TAVI [9]. In our case, we suspect that a change in flow through the aorta or mechanical damage during the TAVI could have led to the dissection of the neointima and aortic thrombosis. Additionally, myocardial infarction in patients after TAVI can be the result of co-existing leaflet thrombosis and can cause difficulty in performing PCI procedures due to impaired coronary access [10]. In our case, the patient’s symptoms were most likely associated with the clinical presentation of ViV thrombosis and severe aortic graft thrombosis, in spite of the administration of SAPT—aspirin. The initial treatment with LMWH converted into VKA oral anticoagulants was sufficient to promote thrombus resolution and prevent recurrence at 3-month follow-up.

## Summary

Aortic graft thrombosis is an extremely rare complication in patients after ViV-TAVI, which may be caused by neointima rupture and can be successfully treated with anticoagulation. The angio-CT may be the alternative for coronary artery assessment in patients with NSTEMI in the presence of aortic graft and valve thrombosis.

## Supplementary Materials

The following supporting information can be downloaded at: https://www.mdpi.com/article/10.3390/diagnostics14182070/s1, Movie S1, dissection of the neointima within Ascending Aortic Prosthesis.
